# Probable Potential Role of Urate Transporter Genes in the Development of Metabolic Disorders

**DOI:** 10.7759/cureus.2382

**Published:** 2018-03-28

**Authors:** Sabitha Vadakedath, Venkataramana Kandi

**Affiliations:** 1 Biochemistry, Chalmeda Anand Rao Institute of Medical Sciences; 2 Department of Microbiology, Prathima Institute of Medical Sciences

**Keywords:** metabolic syndrome, heart diseases, stroke, diabetes, uric acid, urate transporters, genes, insulin resistance, hyperurecaemia, urate transporter genes

## Abstract

Metabolic disorders are a group of interrelated conditions which increases the risk of developing heart diseases, stroke, and diabetes. These usually occur as a consequence of deficiency of enzymes involved in biochemical reactions in the body. The dietary habits, lack of physical exercise, stress, and genetic susceptibility leads to an increased risk of developing metabolic disorders. A diet rich in processed food items containing high calories aggravates the production of a purine metabolite, the uric acid (UA). UA functions as an antioxidant, protects against inflammation, aging, and cancer. It exists as urate ions in the circulation and blood level of UA is maintained by a balance between its production in the liver and its excretion by the renal tubules. The regular excretion of UA through the kidneys is necessary to maintain optimum blood levels of UA (3-7 mg/dl). There are various transporters of uric acid present around the renal tubules, which help in reabsorption of UA into the blood. These urate transporters (UT) are proteins coded in the genes. Mutations in these genes may prompt disturbances in UA reabsorption, and could lead to the development of hyperuricaemia, insulin resistance, endothelial dysfunction, diabetes and other metabolic diseases. This paper reviews eight such genes coding for UTs and attempts to unravel the link between the activities of UA, UTs, and the consequences during mutations in the genes coding for the UTs in the development of metabolic disorders. The genes reviewed included SLC2A9, SLC17A1, SLC22A12, SLC16A9, GCKR, LRRC16A, PDZK1, and ABCG2.

## Introduction and background

Metabolic disorders are a cluster of conditions which may include abdominal obesity, high blood pressure, high blood sugar, high serum triglycerides and low serum high-density lipoprotein (HDL). Metabolic diseases are caused due to imbalances in energy utilization and storage, and is associated with risk of developing chronic diseases like the cardiovascular disease and type 2 diabetes mellitus (T2DM) [1]. Among the several factors which contribute to the development of metabolic disorders, increased uric acid (UA) levels combined with a dietary consumption of fructose was noted to be significant. It was observed that a high intake of fructose in the diet leads not only to hyperuricemia (raised serum UA levels) but also to metabolic disorders, kidney diseases, and cardiovascular diseases [2].

UA is a heterocyclic compound of carbon, nitrogen, hydrogen, and oxygen, and is an oxidation product of purine metabolism present in the blood circulation. UA acts as an antioxidant and helps in defense against inflammation, aging, and cancer. UA exists as urate anion at physiological pH, and these urate ions are impermeable to membrane and require a transporter. UA levels in the body are maintained by a balance between its production and excretion. The major site of urate formation is in the liver and it is excreted in the proximal tubule of the kidneys, where 90% of it is reabsorbed into the blood, facilitated by various transporters. Kidneys play a major role in regulating UA levels and are responsible for 60–70% of UA excretion and the remaining 30–40% of UA is secreted into the intestine where it is acted upon by bacteria and then eliminated from the body [3]. The excretion of UA in kidneys consists of its secretion and absorption. It has been noted that 90% of UA filtered by the kidneys is reabsorbed, and the reabsorption of UA is facilitated by various urate transporters (UT) present around the renal tubules [4]. These UTs are all proteins coded by genes, and any genetic variation in these genes can affect the concentrations of UA and is a risk factor for hyperuricemia (HU) and subsequent complications that include lifestyle disorders like obesity, chronic kidney disease (CKD), cardiovascular disease, gout, T2DM, etc. [5]. Elevated serum UA is an independent predictor of vascular complications like peripheral neuropathy [6].

HU is strongly associated with insulin resistance, an established risk factor for T2DM. It was estimated that for every mg/dl rise in blood UA, the risk of developing T2DM increases by 20% in adults and 15% in children [7]. Hyperuricemia leads to endothelial dysfunction and nitric oxide inhibition, which contributes to insulin resistance and diabetes. Insulin resistance stimulates the urate transporters (URAT1) at renal tubules, promotes reabsorption of UA and results in reduced renal excretion of UA causing HU [8]. Thus, Insulin can influence the actions of UTs. The reabsorption of UA in renal tubules is performed by a set of urate transporters, where some of them associate among themselves by forming a complex of UT. The UTs are proteins coded by genes and help in the transport as well as reabsorption of UA into the renal tubules. The UTs also facilitate the transport of molecules like sodium, chloride, organic acids, glucose, and certain drugs, etc., across the renal tubules. The aim of this article is to systematically review various genes coding for the UTs, their functions and consequences of loss/mutation/polymorphism of the genes. We also focussed on finding the probable link between raised UA and the development of metabolic disorders.

## Review

Solute carrier family 2 member 9 (SLC2A9)

SLC2A9 is a high-affinity urate transporter present in the apical and basolateral membrane of the proximal convoluted tubule (PCT) of the kidneys. It carries information regarding the synthesis of a protein, glucose transporter 9 (GLUT9). Hence, this gene is also commonly called as GLUT9. GLUT9 is involved in reabsorption of hexoses, but pH variations can make this transporter to carry negatively charged purine derivatives such as urate instead of hexoses [9]. Its affinity to reabsorb and transport UA is similar to the activity of URATv1 gene located in the epithelial cells of PCT. Once UA is absorbed, it gets transported from the tubular region into the peritubular spaces, and the uptake from luminal spaces is facilitated by solute carrier family 22 member 12 (SLC22A12) UT gene. GLUT9 reabsorbs and carries UA based on concentration gradient or with the help of voltage driven transporter 1 (URATv1). Pancreatic beta cells sense the blood glucose concentrations and regulate the secretion of insulin. This is done in two phases, the first rapid phase and the second prolonged phase of insulin secretion [10]. GLUT9 is responsible for glucose uptake by the cells in the second phase of insulin secretion. Loss of GLUT9 activity or a mutation leads to raised serum UA levels, increased postprandial glucose levels in blood, and raised insulin secretion. The urate efflux is mediated only by basolateral SLC2A9, and a loss in its function leads to total reabsorption defect [11].

The experimental evidence indicates that SLC2A9 is a direct target gene for p53 (apoptotic gene) and plays a key role in reducing reactive oxygen species (ROS) through uric acid transport. Thus, its expression is regulated by p53 gene and is mediated by oxidative stress [12]. The role of oxidative stress in the development of type 2 diabetes is a known fact. Altered redox potential of the membrane in turn alters protein folding, increases protein degradation and raises serum UA levels in T2DM and other metabolic diseases.

**Figure 1:**
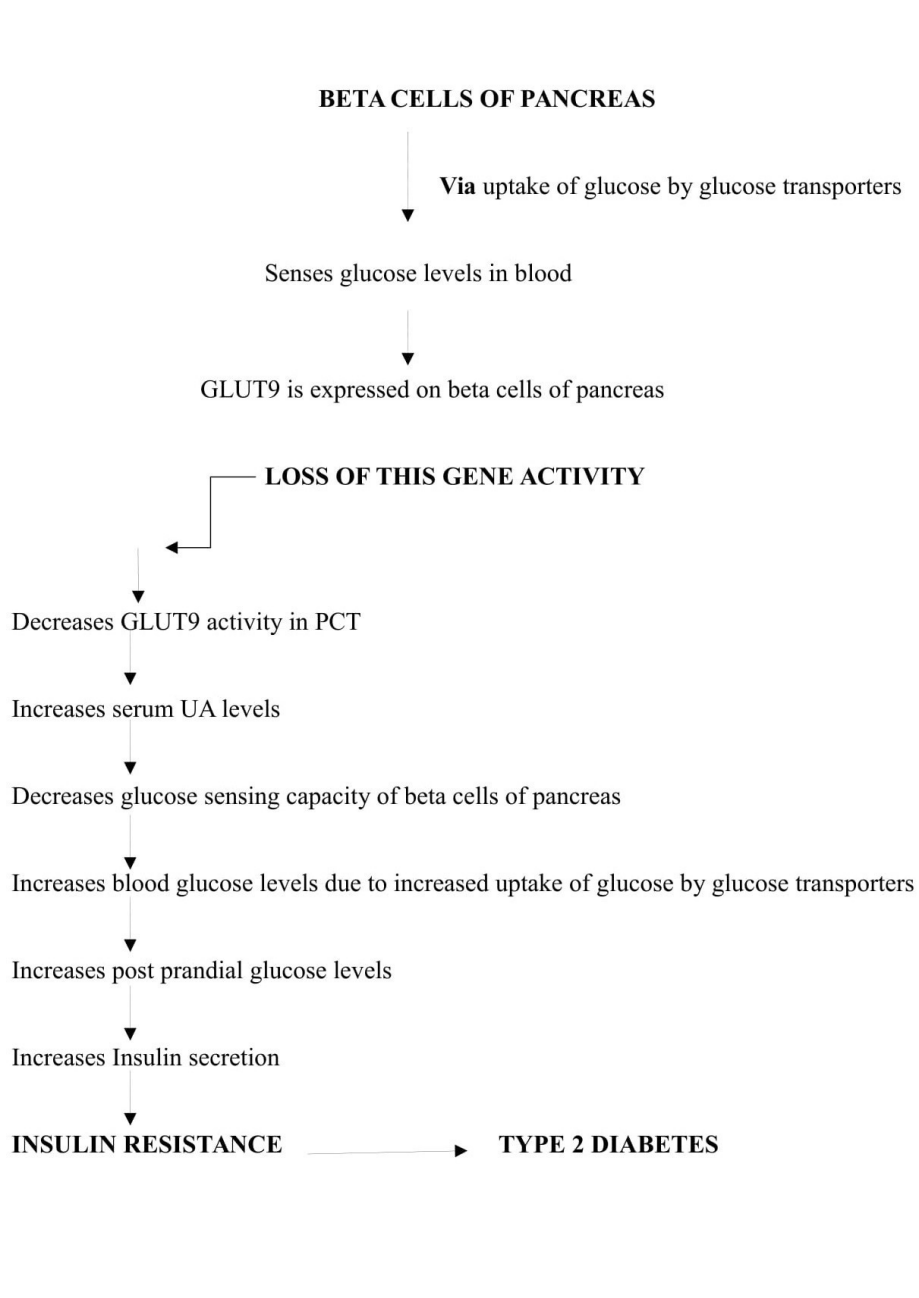
Flow chart showing the role of SLC2A9/GLUT9 gene and consequences of its mutation. GLUT9: Glucose transporter 9; PCT: Proximal convoluted tubule of Kidney; UA: Uric acid.

Mutation in this gene or loss of its activity leads to hyperuricaemia, gout, Alzheimer, and T2DM as shown in Figure 1.

Solute carrier family 17 member 1 (SLC17A1)

SLC17A1 is a family of nine transporters majorly involved in phosphate transport and organic anion transport. SLC17A1 transporter facilitates the transport of phosphate into the cells via sodium co-transport present in the renal brush border membrane [13]. This gene is expressed on apical membrane of proximal convoluted tubules (PCT) and hepatocytes. It helps in phosphate homeostasis by altering membrane potential (sodium). By creating a voltage flux using the chloride ions it pumps organic anions like urate ions into the urine side of tubules. It not only helps in urate excretion through the urine side of the tubules but also aids in inorganic phosphate reabsorption [14]. Hence, it is also called as type 1 sodium-dependent phosphate cotransporter (NPT1).

SLC17A1 also interacts with hepatic nuclear factor 1-alpha (HNF1α) as it is expressed in hepatocytes. The electrogenic nature of this transporter facilitates the clearance of organic anions like urate both from liver and kidneys. It interacts with HNF-1α, which acts as a transcriptional regulator of insulin secretion, bile acid and HDL metabolism, and mutation in SLC17A1 may lead to hypercholesterolemia and hyperglycemia. It was found in experimental studies that this gene activity is regulated by Na^+^/H^+^ exchanger regulatory factor 1 (NHERF1) by inhibiting phosphate uptake by PCT cells, which in turn inhibits NPT1 gene activity. NHERF1 present in kidney regulates NPT1 activity by stimulating phospholipase-C (PLC) and protein kinase C (PKC) signaling molecules. We know that membrane potential changes lead to sequential events in the signaling molecules to release hormone thus, NHERF1 may cause a change in membrane flux which influences insulin secretion via these PLC and PKA [15]. Thus, NPT1 indirectly has a role in insulin secretion during its regulatory process.

**Figure 2:**
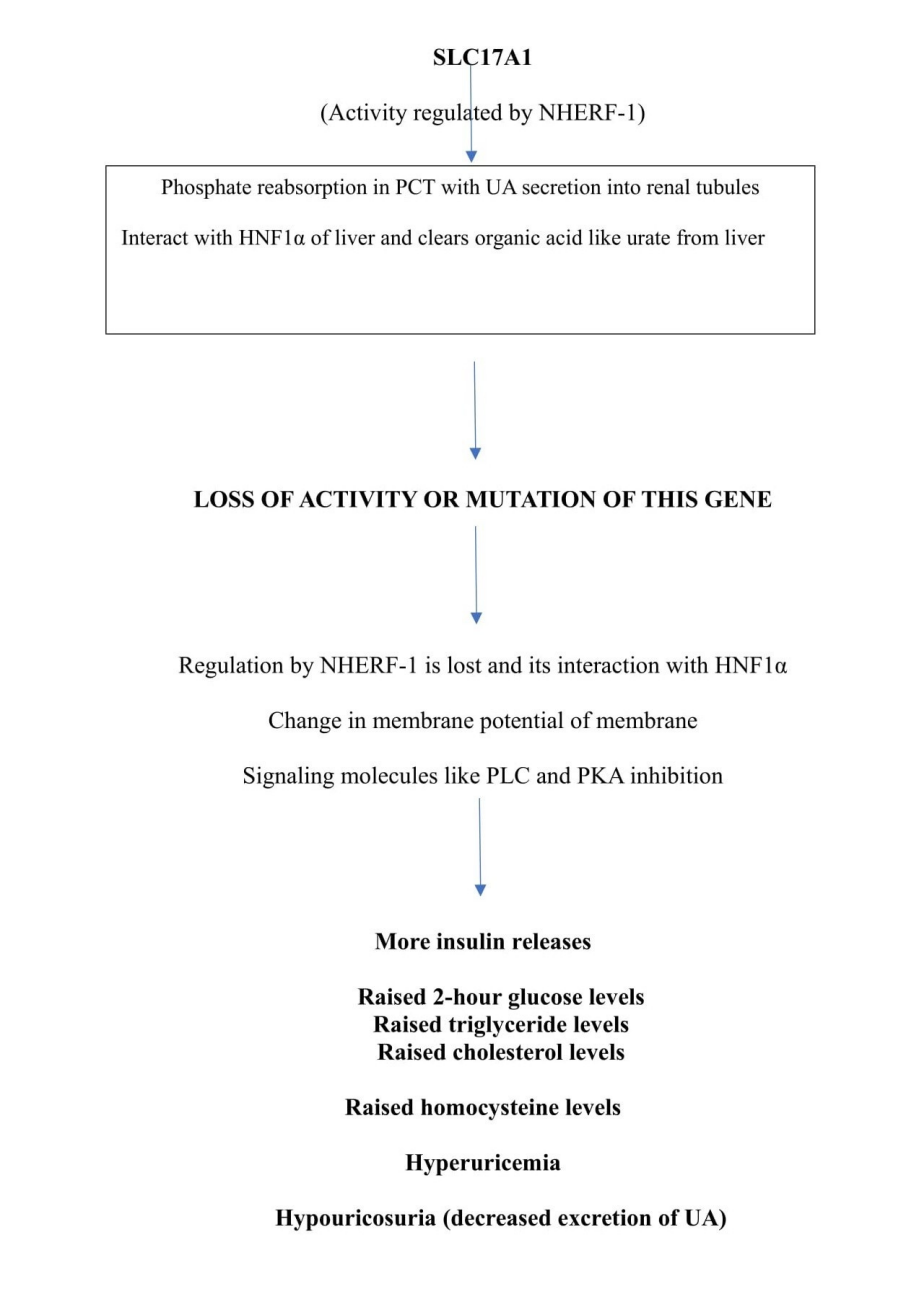
Flow chart showing functions of SLC17A1 gene and consequences of its mutation. NHERF1: Na^+^/H^+^ - exchanger regulatory factor 1; PCT: Proximal convoluted tubule of kidney; HNF1α: Hepatic Nuclear Factor 1 alpha; PLC: Phospholipase-C; PKA: Protein Kinase C.

It was also found that single nucleotide polymorphism (SNP) on SLC17A1 gene or a mutation in this gene not only leads to decreased UA excretion but raises the blood levels of UA leading to hyperuricemia [16]. Hyperuricemia leads to hyper homocystenemia (because UA acts on endothelial function), hypercholesterolemia and raised 2-hour glucose levels. SLC17A1 effects slow phase release of insulin secretion from beta cells of pancreas as shown in Figure 2.

Solute carrier family 22 member 12 (SLC22A12)

Solute carrier family 22 belongs to an organic anion transporter (OAT) family. There are about 10 members of OAT family transporters located at various regions of renal tubules, and these are involved in the transport of various metabolites, drugs, and toxins [17]. Of these 10 OAT family transporters, OAT3 and OAT4 help in reabsorption of UA from urine. SLC22A12 codes for a protein, urate transporter 1 (URAT1), which is a renal urate – anion exchanger found across the apical membrane of PCT. Hence, SLC22A12 is also called as urate anion exchanger 1 (URAT1). It helps in the reabsorption of uric acid from the urine to the PCT in exchange with organic anions like lactate, nicotinic acid, etc. to maintain electrical neutrality. Therefore, it is also called as organic anion transporter 4 like protein (OAT4) [18].

The structural analysis of URAT1 revealed an N-glycosylation site as well as phosphorylation sites which helps in its regulation. The phosphorylation of URAT1 by phosphokinase-C (PKC) stimulates uptake of UA by OAT3 and OAT4, which then is reabsorbed with the help of URAT1 across the PCT of kidney. The PKC phosphorylation can also activate phospho inositol-3- kinase (PI3K) pathway which has a role in insulin secretion (via various signaling molecules) and aids in glucose uptake by glucose transporters. The release of insulin from beta cells of pancreas also increases the reabsorption of UA in PCT. Hence mutation in the gene may alter beta-cell function and raised two-hour glucose levels like SLC2A9 gene. So, it can be concluded that URAT1 and GLUT9 together efficiently help in UA reabsorption in PCT [19]. URAT1 protein interacts with PDZK1 gene in the formation of multimolecular complex ‘transportsome’ that involves co-operation of multiple transporters [20].

**Figure 3:**
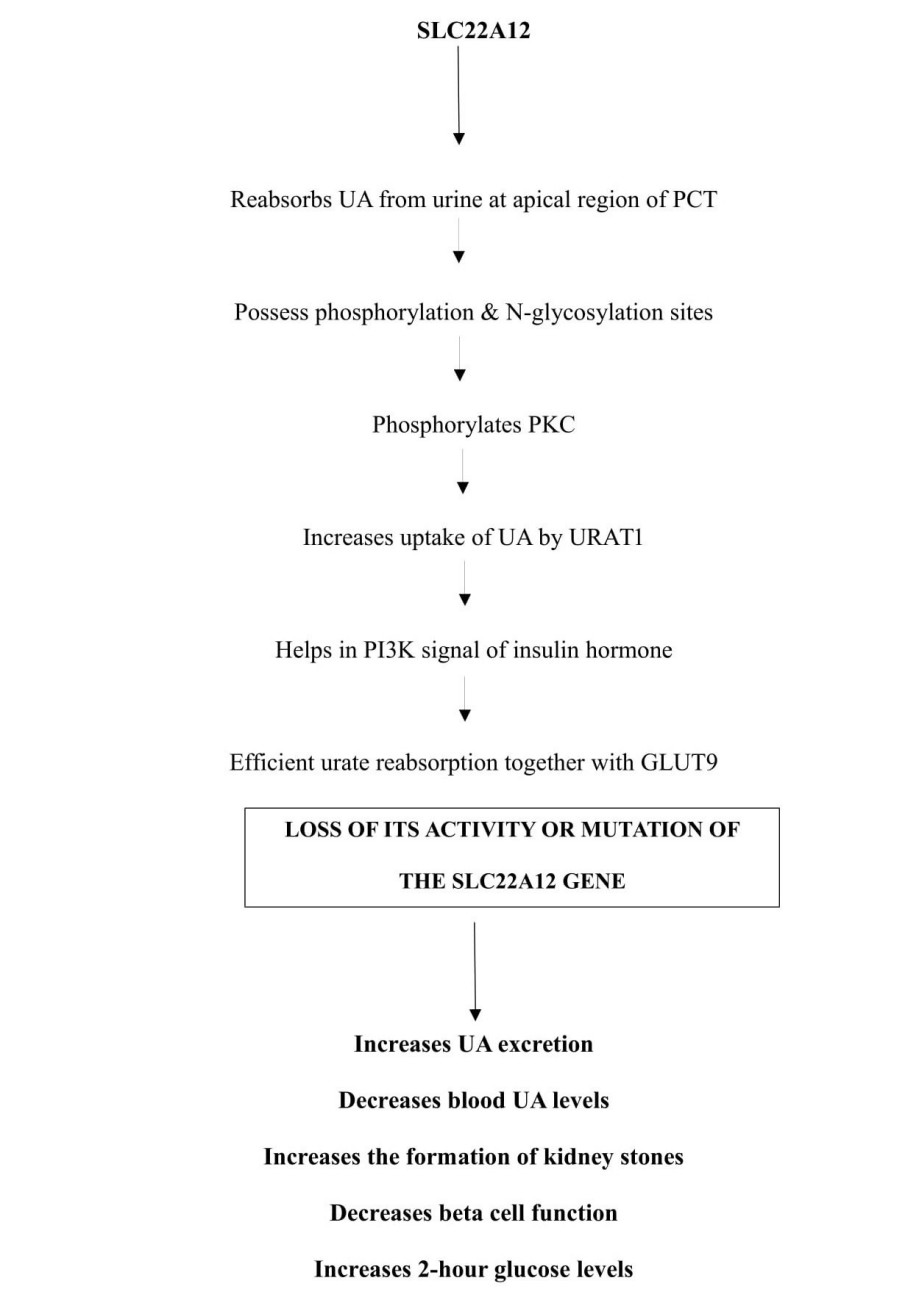
Flow chart showing the functions of SLC22A12 gene and the consequences of its mutation. SLC22A12: Solute carrier family 22; UA: Uric acid; PCT: Proximal convoluted tubules of kidney; PKC: Protein kinase C; URAT1: Urate anion exchanger1; GLUT9: Glucose transporter 9; P13K: Phospho inositol- 3- kinase.

As URAT1 is also involved in the transport of drugs and pharmacological agents, use of anti-inflammatory, hypertensive drugs could increase the excretion of UA, thereby decreasing its levels in the blood [21]. Mutation in this gene leads to idiopathic renal hypouricemia, increased urate excretion, kidney stones, and diabetes as shown in Figure 3.

Solute carrier family 16 member 9 (SLC16A9)

This gene codes for a protein which helps in the transport of monocarboxylic acids like pyruvic acid, lactic acid, etc. Hence, this gene belongs to the family of monocarboxylates which specifically codes for monocarboxylate transporter 9 (MCT9). MCT9 helps in the transport of monocarboxylic acids together with proton-linked transport. The monocarboxylates play a vital role in the cellular and metabolic communications between tissues [22]. MCT9 is expressed in various tissues and in the kidneys. In kidneys, it functions as a sodium-dependent monocarboxylate transporter. It has been observed in experimental studies that MCT9 has a role in carnitine transport. Carnitine is a transporter of lipid molecules across the mitochondrial membrane, which helps in the transport of long-chain fatty acids. Kidneys absorb 95% of carnitine from glomerular filtrate via active sodium-dependent transport mechanism. Carnitine is mostly excreted by glomerulus of kidney and any defect in the re-absorptive function of kidney decreases carnitine excretion. When carnitine is not excreted regularly, it competes with UA for its excretion leading to hyperuricemia [23].

A study by Kolz et al. had noted that there is a triangular association between serum UA, MCT9 and metabolites like carnitine, acylcarnitine, and propionyl carnitine [24]. Therefore, it indicates that MCT9 may be regulating UA levels by creating a balance between its excretion in the intestine and the kidneys. MCT9 is indirectly linked with UA excretion via carnitine. Thus, it helps in the transport of extra renal UA, mostly from the intestine. So, a defective function of kidneys will allow carnitine to compete for UA in the intestinal epithelial cells, leading to renal overload gout. Thus, a block in intestinal excretion of UA raises its level in blood, causing HU. MCT9 acts along with the adenosine triphosphate-binding cassette sub-family G member 2 protein (ABCG2) gene that also has a role in excretion of UA from the intestine [25]. Kidney dysfunction not only leads to gout but also to cardiovascular diseases as carnitine levels are decreased, which causes increased low-density lipoprotein (LDL) activities. Carnitine-related products like acetyl carnitine have a role in improving insulin-mediated glucose disposal in healthy individuals and in T2DM patients. Acetyl carnitine has a role in compensating energy demands by regulating the availability of acetyl and acyl derivatives, which in turn control the synthesis of key glycolytic and gluconeogenic enzymes [25].

**Figure 4:**
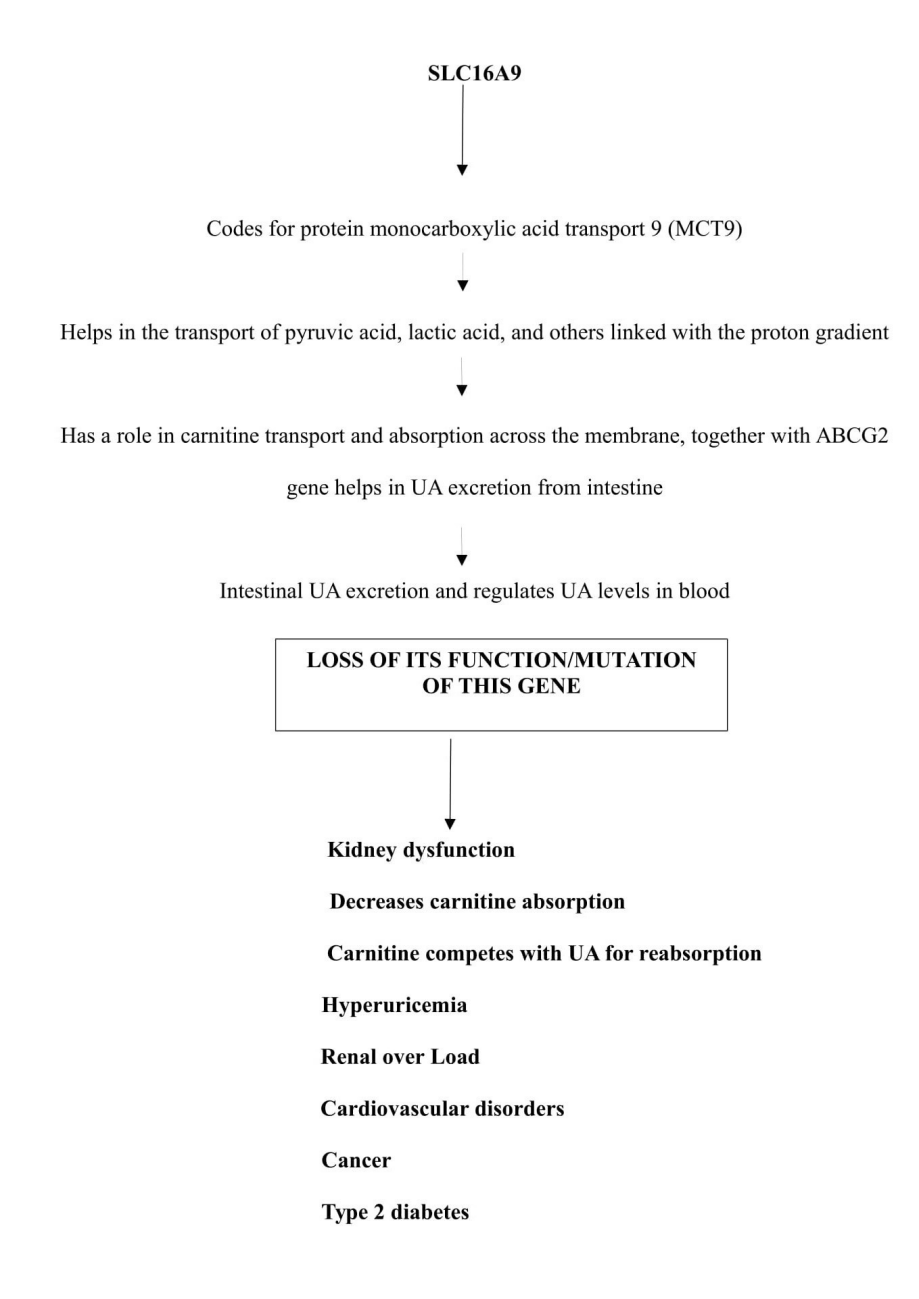
Flow chart showing SLC16A9 gene function and the consequences of its mutation. SLC16A9: Solute carrier family 16 member 9; ABCG2: Adenosine triphosphate binding cassette sub-family G member 2 protein; UA: Uric acid.

Mutation or loss of this gene activity leads to gout of extra renal origin, obesity, cardiovascular complications, kidney disorders, T2DM, and cancer as shown in Figure 4.

Glucokinase regulatory protein (GCKR)

This gene codes for a protein glucokinase (GK), which is produced in hepatocytes. The glucokinase is an enzyme which helps in phosphorylation of glucose at C6 position and its activity is dependent on blood glucose levels. It acts as a glucose sensor in response to raised blood glucose levels. GK enzyme moves between nucleus and cytosol, i.e., at high blood glucose levels the GK enzyme moves to cytosol, and forms a complex with microfilaments of hepatocytes and then phosphorylates available glucose molecules in conjugation with the insulin hormone function [26]. The glucose sensor activity of GK enzyme is due to its low affinity for glucose, positive co-operativity (once glucose molecules are phosphorylated the formed product glucose 6 phosphate makes the rest of the glucose molecules to get phosphorylated by GK) and lack of product inhibition (i.e., glucose 6 phosphate will not inhibit the action of the enzyme GK) [27].

Depending on the ATP (adenosine triphosphate) levels of the cell, the glucose 6 phosphate enters a glucose oxidative pathway (hexose monophosphate shunt (HMP) pathway) to produce pentoses, and nicotinamide adenine dinucleotide phosphate (NADPH). Pentoses are utilized in nucleic acid metabolism, and the availability of ribose-5-phosphate increases UA levels. NADPH is used for the biosynthesis of fat (fatty acid synthesis, cholesterol synthesis). Alternatively, during fasting or energy-deprived state, GK is inactive and thus metabolic pathway proceeds for gluconeogenesis and inactivation of GK during gluconeogenesis prevents a futile cycle (energy of the cell is dissipated as heat) [28]. Its expression in beta cells of pancreas regulates insulin-dependent glucose secretion by modulating ATP/ADP ratio. In the hepatocytes, GK helps in the disposal of glucose by converting it into glycogen [29].

**Figure 5:**
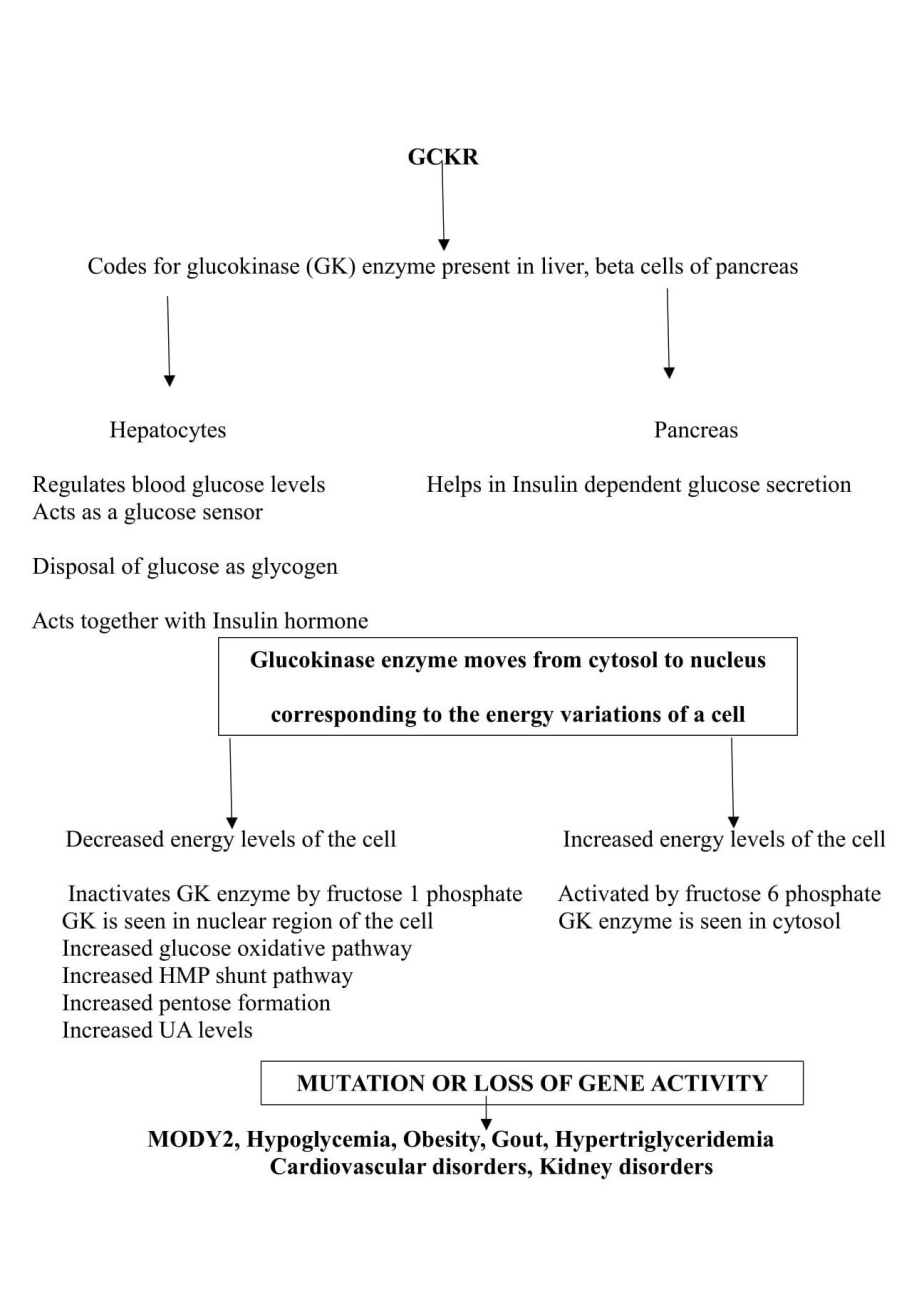
Flow chart showing GCKR gene function and the consequences of its mutation. GCKR: Glucokinase regulatory protein; HMP: Hexose monophosphate shunt; MODY2: Maturity onset diabetes of the young2; UA: Uric acid.

Mutation in this gene leads to hypoglycemia, hypertriglyceridemia, obesity, and cardiovascular risk. Loss of its activity leads to the development of hyperglycemia and maturity-onset diabetes of the young 2 (MODY2). Enhanced activity of HMP pathway increases the availability of pentoses, which in turn leads to gout and kidney diseases as shown in Figure 5 [30].

Leucine-rich repeat-containing protein 16 A (LRRC16A)

This gene encodes a protein called capping protein ARP2/3 and myosin-I linker (CARMIL), which is an inhibitor of actin capping protein (CP). The CP is an essential element of actin cytoskeleton, which binds to the actin filaments and prevents its polymerization (process of converting monomer to polymer). The CP inhibits polymerization of actin filaments by binding to its barbed ends. CARMIL protein is expressed by various tissues, but predominantly in kidneys. In the apical membrane of proximal tubules, urate transportsome (UTS) is formed. UTS is formed by several UA transporter proteins like URAT1, OAT4L, NPT1, and ABCG2 which have a role in the absorption of UA in exchange with sodium ions (Na^+^). The UTS also interacts with scaffolding proteins like PDZK1, NHERF1, and cytoskeletal filaments like actin [31].

**Figure 6:**
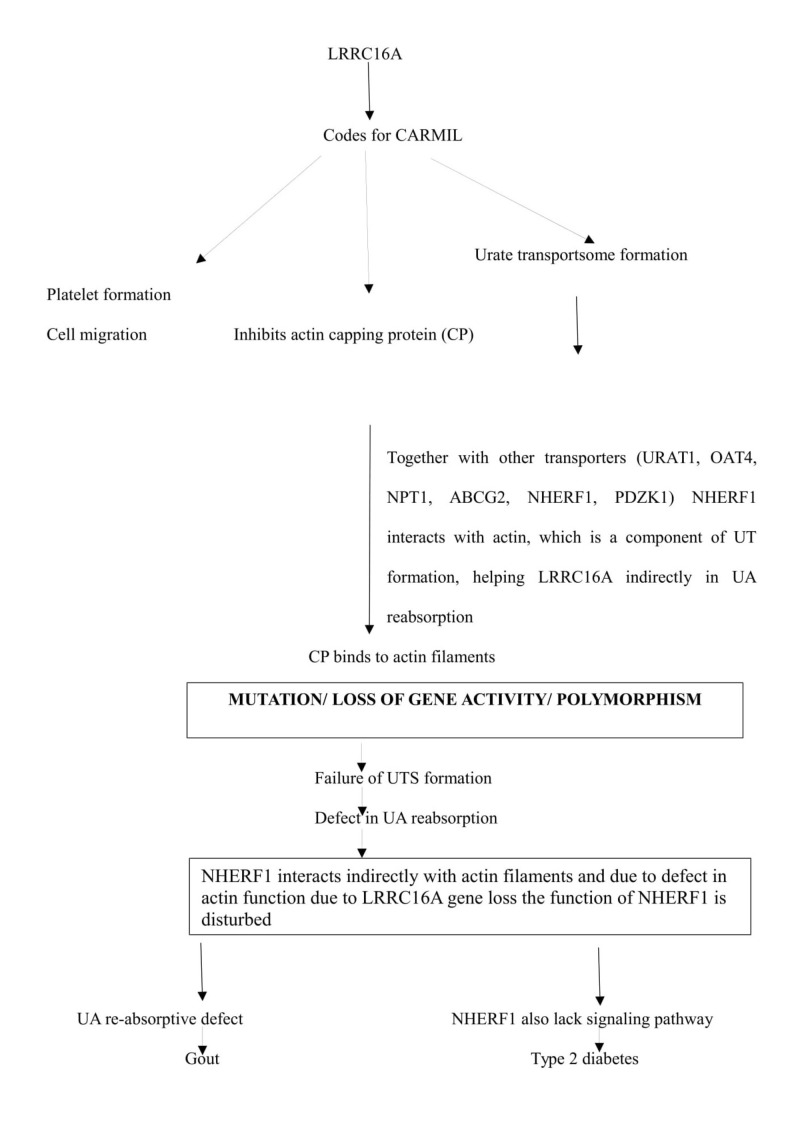
Flow chart showing LRRC16A gene function and the consequences of its mutation. LRRC16A: Leucine rich repeat containing protein 16 A; CARMIL: Capping protein ARP2/3 and myosin-I linker; URAT1: Urate transporter 1; OAT4: Organic anion transporter 4; NPT1: Sodium dependent phosphate cotransporter 1; ABCG2: Adenosine triphosphate binding cassette sub-family G member 2 protein; NHERF1: Na^+^/H^+^ - exchanger regulatory factor 1; PDZK1: Protein domain containing 1; UT: Urate transporter; UTS: Urate transportsome.

Thus, CARMIL activity in UTS is essential. Mutation or loss of gene activity or polymorphism leads to operative defect in UTS activity, spindle fiber formation, and cell migratory defect. During UTS formation NHERF1 interacts with actin filaments and can inhibit NPT1 activity, by aggravating phospholipase C (PLC) and protein kinase C (PKC) signaling molecules. Considering the fact that Insulin hormone is regulated mainly by these signaling molecules, a defect in the gene coding for CARMIL could be associated with gout as shown in Figure 6 [32, 33].

Protein domain containing 1 (PDZK1)

This gene codes for a scaffolding protein. A scaffolding protein connects membrane proteins and regulatory components, and also regulates its expression on the surface of epithelial cells. PDZK1 protein is expressed on the epithelial cells of kidneys, liver, pancreas, gastrointestinal tract, and adrenal cortex. It acts as a scaffolding protein by interacting with membrane-associated proteins like the MAP-17, and with other cytoplasmic proteins. Hence, PDZK1 has a role in cell proliferation, differentiation and ion transport [34].

It was found in experimental studies that PDZK1 increases the expression of the Na^+^/H^+^ exchange regulatory factor 3 (NHERF-3) in the brush border cells of renal tubules. PDZK1 and NHERF 3 together act as scaffolding protein [35-36]. Along with Na/Pi solute carrier, NHERF3 helps in the transport of phosphate, chloride, different organic anions like UA, and drugs like probenecid [37]. Another scaffolding protein NHERF1 interacts with PDZK1, and activates NHERF3 and CFEX transporters, which play a crucial role in renal handling of bicarbonate and NaCl homeostasis [38]. PDZK1 also potentiates chloride channel activity of cystic fibrosis conductance regulator (CFTR), and compartmentalizes signaling protein kinase A (PKA) [39-40].

PDZK1 organizes a transportsome and regulates several transporter functions like urate and drug transport. A urate transportsome is formed by linking several UA transporters like ABCG2, SLC22A12, NPT1, which in turn relate to each other by scaffolding proteins like PDZK1, NHERF1 at the apical surface of renal tubules. Mutations in these UA transporters (ABCG2, SLC22A12, NPT1) also leads to hyperuricemia and gout. So, loss of activity of PDZK1 indirectly causes gout [41].

**Figure 7:**
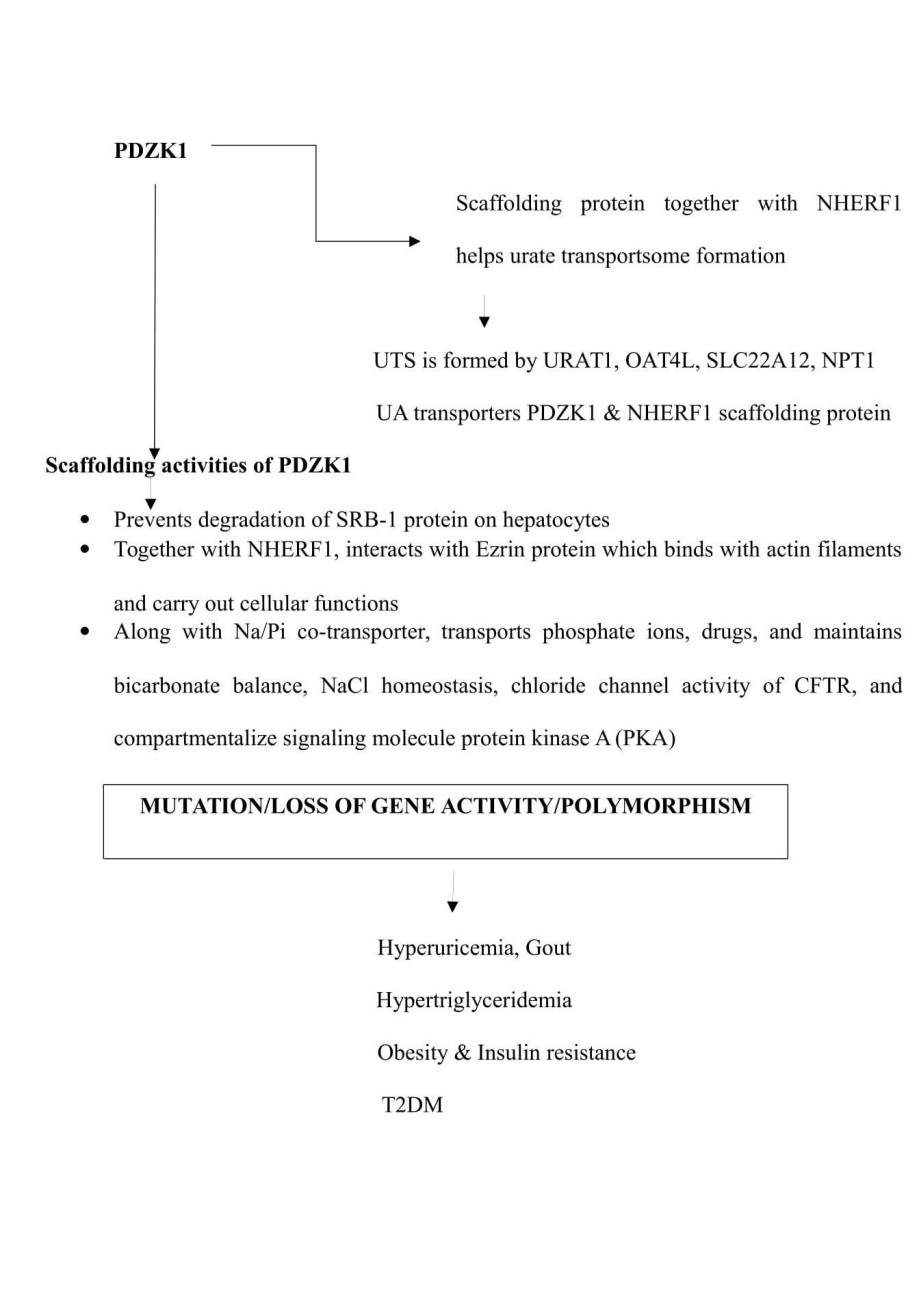
Flow chart showing function of the gene PDZK1 and the consequences of its mutation. PDZK1: Protein domain containing 1; NHERF1: Na^+^/H^+^ - exchanger regulatory factor 1; UTS: Urate transportsome; URAT1: Urate transporter 1; OAT4: Organic anion transporter 4; SLC22A12: Solute carrier family 22A12; NPT1: Sodium dependent phosphate cotransporter 1; UA: Uric acid; SRB-1: Scavenger receptor class B type 1; NaPi: Sodium phosphate; NaCl: Sodium chloride; CFTR: Cystic fibrosis conductance regulator; PKA: Protein kinase A; T2DM: Type 2 diabetes mellitus.

PDZK 1 also stabilizes and prevents the degradation of scavenger receptor class B type 1 (SRB-1) protein on the surface of hepatocytes and endothelial cells. SRB 1 is a cell surface glycoprotein that binds to HDL with high affinity. It also plays an important role in reverse cholesterol transfer function of HDL, and has a role in atheroprotection [42]. PDZK1 and NHERF1 together act as a scaffolding protein in urate transportsome formation. NHERF1 along with adaptor protein ezrin functions as signal transducers between cell membrane and actin cytoskeleton. Actin and associated cytoskeletal protein, together with scaffolding proteins, regulate receptor signaling to maintain cellular homeostasis. PDZK 1 interacts indirectly with LRCC16A gene due to cooperative function of cytoskeletal proteins as shown in Figure 7 [43].

Adenosine triphosphate-binding cassette sub-family G member 2 protein (ABCG2)

ABCG2 gene codes for ATP binding cassette (ABC) transporter. ABC transporters utilize energy by hydrolyzing ATP and transports substrates across cell membrane [44]. ABCG2 was initially found to be a xenobiotic transporter in a multi-drug resistant human breast cancer cell types [45]. As it was identified in breast tissue it is also called as breast cancer resistance protein (BCRP). It also protects the cell against the actions of chemotherapeutic drugs like anthracycline and mitoxantrone exposure (cellular defense). ABCG2 found in placenta protects the fetus against the xenobiotic substances present in the maternal circulation. The drug blocking action of this gene is due to its ability to inhibit the absorption of drugs at the intestinal surface, blood-brain barrier, and blood-testis barrier [46]. It also has a role in the secretion of vitamins like riboflavin and biotin into the breast milk.

**Figure 8:**
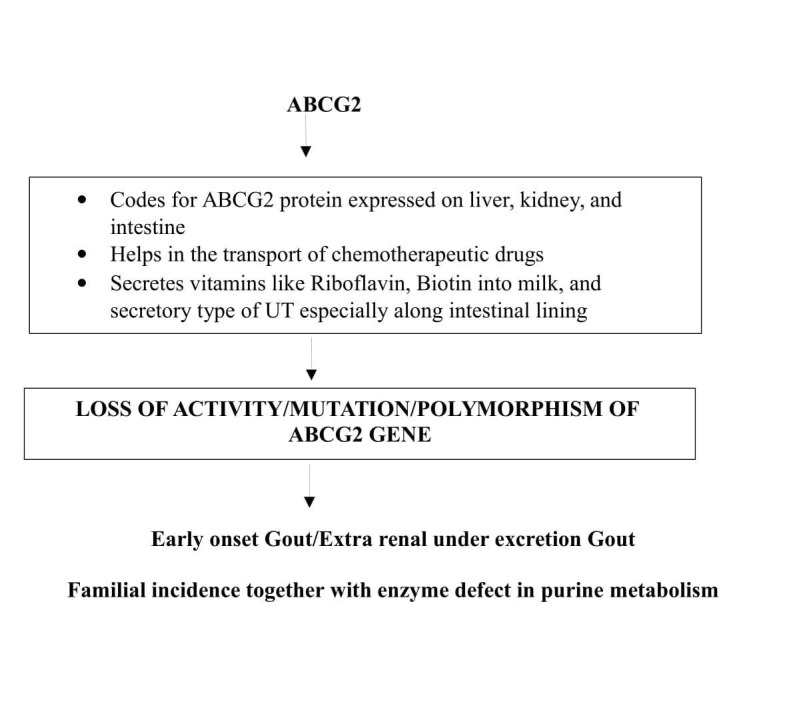
Flow chart showing ABCG2 functions and the consequences of its mutation. ABCG2: Adenosine triphosphate binding cassette sub-family G member 2 protein; UT: Urate transporter.

ABCG2 is an efflux urate transporter seen on the proximal renal tubules. It is a high capacity secretory urate transporter (i.e., secretes UA into renal tubules) whose activity is completely dependent on intracellular levels of UA. ABCG2 is also expressed in liver and intestinal cells. Experimental studies on mice have confirmed that dysfunction or loss of activity of ABCG2 increases the urinary excretion of UA due to the action of URAT1 on intestine. This concludes that decreased urate excretion could be via extra-renal pathway. Thus, ABCG2 regulates UA levels via the extra-renal UA excretion, which is the major cause of extra-renal underexcretion, and gout [47]. Later it was confirmed that ABCG2 dysfunction in renal tubules may lead to early onset gout which has a familial background. An enzymatic defect in purine metabolism like hypoxanthine-guanine phosphoribosyltransferase (HGPRTase) leading to Lesch–Nyhan syndrome, super activity of phosphoribosyl pyrophosphate synthetase enzyme (PRPP synthetase), familial juvenile hypouricemic nephropathy may also contribute to a defective function of ABCG2 gene or protein [48-50]. The role of this gene in T2DM may be associated with its regulation by PIP (PI3K)/AKT pathway (phospho inositol phosphate/protein kinase pathway) as shown in Figure 8.

## Conclusions

Uric acid is a product of purine metabolism and its reabsorption majorly happens in the renal tubules. There are about eight genes coding for urate transporters involved in its reabsorption process present in various parts of renal tubules. These transporters are arranged in a pocket-like or a scaffolding-type structure around the renal tubules, and the genes coding for them acts in combination to transport the urate and other molecules. Mutation or polymorphism or loss of genes coding for transport causes hyperuricemia, which increases the glycation of proteins, and later could be responsible for the development of metabolic disorders like T2DM. Hence, identification of mutations in the genes encoding urate transporters will play a crucial role in the control and prevention of metabolic disorders.

## References

[1] Kaur J (2014). A comprehensive review on metabolic syndrome. Cardiol Res Pract.

[2] Hallfrisch J (1990). Metabolic effects of dietary fructose. FASEB.

[3] Bobulescu IA, Moe OW (2012). Renal transport of uric acid: evolving concepts and uncertainties. Adv Chronic Kidney Dis.

[4] Bo S, Cavallo-Perin P, Gentile L, Repetti E, Pagano G (2001). Hypouricemia and hyperuricemia in type 2 diabetes: two different phenotypes. Eur J Clin invest.

[5] Dong Z, Zhou J, Jiang S (2017). Effects of multiple genetic loci on the pathogenesis from serum urate to gout. Sci Rep.

[6] Facchini F, Chen YD, Hollenbeck CB, Reaven GM (1991). Relationship between resistance to insulin-mediated glucose uptake, urinary uric acid clearance, and plasma uric acid concentration. JAMA.

[7] Bhole V, Choi JW, Kim SW, de Vera M, Choi H (2010). Serum uric acid levels and the risk of type 2 diabetes: a prospective study. Am J Med.

[8] Bandaru P, Shankar A (2011). Association between serum uric acid levels and diabetes mellitus. Int J Endocrinol.

[9] Augustin R, Carayannopoulos MO, Dowd LO, Phay JE, Moley JF, Moley KH (2004). Identification and characterization of human glucose transporter-like protein-9 (GLUT9): alternative splicing alters trafficking. J Biol Chem.

[10] Anzai N, Ichida K, Jutabha P (2008). Plasma urate level is directly regulated by a voltage-driven urate efflux transporter URATv1 (SLC2A9) in humans. J Biol Chem.

[11] Dinour D, Gray NK, Campbell S (2010). Homozygous SLC2A9 mutations cause severe renal hypouricemia. J Am Soc Nephrol.

[12] Itahana Y, Han R, Barbier S, Lei Z, Rozen S, Itahana K (2015). The uric acid transporter SLC2A9 is a direct target gene of the tumor suppressor p53 contributing to antioxidant defense. Oncogene.

[13] Reimer RJ (2013). SLC17: a functionally diverse family of organic anion transporters. Mol Aspects Med.

[14] Iharada M, Miyaji T, Fujimoto T, Hiasa M, Anzai N, Omote H, Moriyama Y (2010). Type 1 sodium-dependent phosphate transporter (SLC17A1 Protein) is a Cl(-)-dependent urate exporter. J Biol Chem.

[15] Cheret C, Doyen A, Yaniv M, Pontoglio M (2002). Hepatocyte nuclear factor 1 α controls renal expression of the Npt1-Npt4 anionic transporter locus. J Mol Biol.

[16] Koyama T, Matsui D, Kuriyama N (2015). Genetic variants of SLC17A1 are associated with cholesterol homeostasis and hyperhomocysteinaemia in Japanese men. Sci Rep.

[17] Wheeler D, Garrido JL, Bisello A, Kim YK, Friedman PA, Romero G (2008). Regulation of PTH1R dynamics, traffic and signaling by the Na+/H+ exchanger regulatory factor-1 (NHERF1) in rat osteosarcoma ROS 17/2.8 cells. Mol Endocrinol.

[18] Hediger MA, Johnson RJ, Miyazaki H, Endou H (2005). Molecular physiology of urate transport. Physiology (Bethesda).

[19] Enomoto A, Kimura H, Chairoungdua A (2002). Molecular identification of a renal urate anion exchanger that regulates blood urate levels. Nature.

[20] Boucher J, Kleinridders A, Kahn CR (2014). Insulin receptor signaling in normal and insulin-resistant states. Cold Spring Harb Perspect Biol.

[21] Sun X, Zhang R, Jiang F (2015). Common variants related to serum uric acid concentrations are associated with glucose metabolism and insulin secretion in a Chinese population. PLoS One.

[22] Halestrap AP, Wilson MC (2012). The monocarboxylate transporter family--role and regulation. IUBMB Life.

[23] Loots DT, Mienie LJ, Bergh JJ, Van der Schyf CJ (2004). Acetyl-L-carnitine prevents total body hydroxyl free radical and uric acid production induced by 1-methyl-4-phenyl-1,2,3,6-tetrahydropyridine (MPTP) in the rat. Life Sci.

[24] Matsuo H, Nakayama A, Sakiyama M (2014). ABCG2 dysfunction causes hyperuricemia due to both renal urate underexcretion and renal urate overload. Sci Rep.

[25] Mingrone G (2004). Carnitine in type 2 diabetes. Ann N Y Acad Sci.

[26] Parry MJ, Walker DG (1966). Purification and properties of adenosine 5′-triphosphate-D-glucose 6-phosphotransferase from rat liver. Biochem J.

[27] Niemeyer H, de la Luz Cárdenas M, Rabajille E, Ureta T, Clark-Turri L, Peñaranda J (1975). Sigmoidal kinetics of glucokinase. Enzyme.

[28] van Schaftingen E, Vandercammen A, Detheux M, Davies DR (1992). The regulatory protein of liver glucokinase. Adv Enzyme Regul.

[29] Iynedjian PB (2009). Molecular physiology of mammalian glucokinase. Cell Mol Life Sci.

[30] Suhre K, Shin S-Y, Petersen A-K (2011). Human metabolic individuality in biomedical and pharmaceutical research. Nature.

[31] Sagen JV, Odili S, Bjorkhaug L (2006). From clinic genetic studies of maturity-onset diabetes of the young to unraveling complex mechanisms of glucokinase regulation. Diabetes.

[32] Yang C, Pring M, Wear MA, Huang M, Cooper JA, Svitkina TM, Zigmond SH (2005). Mammalian CARMIL inhibits actin filament capping by capping protein. Dev Cell.

[33] Wright AF, Rudan I, Hastie ND, Campbell H (2010). A 'complexity' of urate transporters. Kidney Int.

[34] Sakiyama M, Matsuo H, Shimizu S (2014). A common variant of leucine-rich repeat-containing 16A (LRRC16A) gene is associated with gout susceptibility. Human Cell.

[35] Kocher O, Comella N, Tognazzi K, Brown LF (1998). Identification and partial characterization of PDZK1: a novel protein containing PDZ interaction domains. Lab Invest.

[36] Shenolikar S, Weinman EJ (2001). NHERF: targeting and trafficking membrane proteins. Am J Physiol Renal Physiol.

[37] Busch AE, Schuster A, Waldegger S (1996). Expression of a renal type I sodium/phosphate transporter (NaPi-1) induces a conductance in Xenopus oocytes permeable for organic and inorganic anions. Proc Natl Acad Sci U S A.

[38] Knauf F, Yang C-L, Thomson RB, Mentone SA, Giebisch G, Aronson PS (2001). Identification of a chloride-formate exchanger expressed on the brush border membrane of renal proximal tubule cells. Proc Natl Acad Sci U S A.

[39] Moyer BD, Duhaime M, Shaw C (2000). The PDZ-interacting domain of cystic fibrosis transmembrane conductance regulator is required for functional expression in the apical plasma membrane. J Biol Chem.

[40] Gisler SM, Pribanic S, Bacic D (2003). PDZK1: I. A major scaffolder in brush borders of proximal tubular cells. Kidney Int.

[41] Higashino T, Matsuo H, Sakiyama M (2016). Common variant of PDZ domain containing 1 (PDZK1) gene is associated with gout susceptibility: a replication study and meta-analysis in Japanese population. Drug Metab Pharmacokinet.

[42] Yesilaltay A, Kocher O, Pal R, Leiva A, Quiñones V, Rigotti A, Krieger M (2006). PDZK1 is required for maintaining hepatic scavenger receptor, class B, type I (SR-BI) steady state levels but not its surface localization or function. J Biol Chem.

[43] Pollard TD, Cooper JA (2009). Actin, a central player in cell shape and movement. Science.

[44] Jones PM, George AM (2004). The ABC transporter structure and mechanism: perspectives on recent research. Cell Mol Life Sci.

[45] Doyle LA, Yang W, Abruzzo LV, Krogmann T, Gao Y, Rishi AK, Ross DD (1998). A multidrug resistance transporter from human MCF-7 breast cancer cells. Proc Natl Acad Sci U S A.

[46] Vlaming ML, Lagas JS, Schinkel AH (2009). Physiological and pharmacological roles of ABCG2 (BCRP): recent findings in Abcg2 knockout mice. Adv Drug Deliv Rev.

[47] Ichida K, Matsuo H, Takada T (2012). Decreased extra-renal urate excretion is a common cause of hyperuricemia. Nat Commun.

[48] Torres RJ, Puig JG (2007). Hypoxanthine-guanine phosphoribosyl transferase (HGPRT) deficiency: Lesch-Nyhan syndrome. Orphanet J Rare Dis.

[49] Becker MA, Meyer LJ, Wood AW, Seegmiller JE (1973). Purine overproduction in man associated with increased phosphoribosylpyrophosphate synthetase activity. Science.

[50] Hart T, Gorry M, Hart P (2002). Mutations of the UMOD gene are responsible for medullary cystic kidney disease 2 and familial juvenile hyperuricaemic nephropathy. J Med Genet.

